# Introduction to the Special Series: National Drug Abuse Treatment Clinical Trials Network and the Opioid Use Disorder Care Continuum—20 years of research informing practice

**DOI:** 10.1186/s13722-022-00291-9

**Published:** 2022-02-02

**Authors:** Andrew J. Saxon, E. Jennifer Edelman

**Affiliations:** 1 Department of Psychiatry and Behavioral Sciences, University of Washington School of Medicine and Center of Excellence in Substance Addiction Treatment and Education, VA Puget Sound Health Care System, 1660 S. Columbian Wy., Seattle, WA 98108 USA; 2 Department of Internal Medicine, Yale School of Medicine, New Haven, CT USA

The special series devoted to research on opioid use disorder (OUD) conducted in the National Institute on Drug Abuse Treatment Clinical Trials Network (CTN) and published in *Addiction Science and Clinical Practice* highlights the tremendous progress that the CTN has made over the past two decades in understanding and improving treatment for OUD. In addition, the series underscores the daunting challenges we still face and the new knowledge we need to gain to deliver high quality, patient-centered, equitable and inclusive treatment to everyone who needs it in the setting they prefer.

The paper by Tai et al. [1] summarizes the history and operations of the CTN, points out the shift in focus from studying OUD treatment solely in specialty substance use disorder treatment programs during the CTN’s early years to its nimble pivot to studying OUD treatment in a variety of health care settings including primary care and specialty care, the Emergency Department, and inpatient hospital wards. This paper also makes the point that we need to learn better how to leverage the electronic medical record and digital technologies to improve OUD treatment further. Some of these themes find amplification in other papers in this series.

Picking up on the CTN’s history, Shulman et al. [2] summarize prior published CTN studies regarding treatment with medication for OUD (MOUD). They describe the CTN’s examination of opioid withdrawal using buprenorphine and buprenorphine treatment of adolescents and young adults, comparison of buprenorphine to methadone regarding their effects on liver health, evaluation of the impact of adding counseling to physician management of OUD with buprenorphine, and a direct comparison of buprenorphine to extended-release naltrexone for treatment of OUD. One consistent piece of emergent knowledge, consonant with other research in the field, is that patients do well when taking MOUD but often do not remain on it long enough and/or only get MOUD intermittently. This background provides the impetus for a next phase CTN study which will use a complex, multisite, randomized design to show the field which approaches to buprenorphine (i.e., standard dose, high dose, or long-acting injectable buprenorphine) and extended-release naltrexone treatment promote the best treatment retention. This study will then follow stable patients who elect to discontinue off MOUD to help determine what, if any, variables might predict ongoing successful abstinence after discontinuing medications. The study will also explore the use of mobile applications in the delivery of behavioral interventions.

On the theme of improving access to and retention on MOUD in primary care, Campbell et al. [3] present the protocol for a CTN study that will test the effectiveness of different strategies for placing a nurse care manager as part of team-based care (the “Massachusetts Model”) in primary care clinics to enhance MOUD provision and will look at the impact of MOUD provision on utilization of health system resources in a hybrid type III cluster randomized design. de la Cruz et al. [4] took another approach to enhancing MOUD delivery in primary care by creating an algorithm for providing buprenorphine treatment that can be incorporated into the electronic medical record which could be tested in the future to assist primary care providers in managing patients on this medication. Hser et al. [5] focus on targeting rural practices for implementation of MOUD in primary care, using telemedicine and describe some early lessons learned from a feasibility project. Just as the project started, the COVID-19 pandemic drastically altered the use of telemedicine such that it became a prevalent mode of primary care delivery with practices scrambling to establish platforms to make it available to their patients. Some possible barriers attributed to the use of telemedicine include difficulties of screening for OUD in primary care, low rates of referral to telemedicine, poor access to broadband and digital devices in rural areas, and questions about insurance coverage for telemedicine.

Turning to another important setting, McCormack et al. [6], recognizing the need for and the complexity of starting buprenorphine treatment at the moment patients with OUD present to an Emergency Department, delineate the framework for a comprehensive implementation project in under-resourced urban and rural Emergency Departments. The work includes both qualitative and quantitative data collection to assess the needs and views of providers and patients, external facilitation, and training to bring Emergency Department staff to readiness to prescribe MOUD, and an ultimate determination of number of patients treated.

Combining the themes of Emergency Department setting and use of technology, Hawk et al. [7] look at the feasibility of using a mobile application to help track outcomes for patients started on buprenorphine in the Emergency Department. Even in an urban setting familiar themes of poor internet access, limited digital skills among patients, and intricacies associated with the application interfered with the majority of patients completing follow-up surveys, though many did complete surveys successfully.

Several studies focused on MOUD delivery both in diverse settings and to diverse populations—a key strategy for which we need to build capacity. Critchley et al. [8] report on individuals with human immunodeficiency virus (HIV) and find, not surprisingly, that individuals with HIV and opioids as their primary drug problem were less likely than individuals with HIV and no substance use problem to engage in HIV primary care. This finding speaks to the need to ensure that persons with HIV and problem opioid use get HIV treatment and MOUD across different settings, including the hospital [9], as a way to halt these twin epidemics.

Because OUD often first manifests in adolescence or early adulthood, Bagley et al. [10] determined the rates of OUD among youth attending six large primary practices. They found an overall OUD rate of 0.7%, that youths with OUD have extensive co-morbidity, and that far less than 50% of these youths receive MOUD in primary care. These important findings speak to the need for us to ascertain and implement robust OUD prevention interventions among children and to intervene early and aggressively with prescribing MOUD to youths with OUD in an effort to avoid a lifetime of disability from this potentially lethal, but very treatable disorder.

Addressing one of the pressing exigencies that society as a whole must grapple with, Burlew et al. [11] call for urgent attention to the inequities across racialized and ethnic groups that also pervade the treatment of and research on OUD and other substance use disorders. They recommend recruiting sufficient numbers of minority participants into OUD research, using measurements appropriate to the racialized and ethnic groups being studied, maintaining awareness of the interplay between social determinants of health and racial and ethnic identity, understanding and accounting for within-race differences, and not lumping non-white participants together when analyzing data. Furthermore, they argue persuasively that we must increase the numbers of racial and ethnic minority investigators as one way to infuse the planning and conduct of OUD research with awareness of the specific treatment needs of racial and ethnic minority populations. The critical guidance in this paper provides an important roadmap for investigators and future work.

Finally, as noted in passing above, we cannot ignore the fact that this series, while planned prior to any inkling of the appearance of a worldwide pandemic, was published at the height of the COVID-19 pandemic which colors every aspect of research on and treatment of OUD. Cowan et al. [12] make a major contribution by conceptualizing the myriad of ways that this pandemic alters the landscape of OUD and the lives of patients and will require a refocus by everyone involved.

In summary, this special series of papers on OUD from the CTN captures much of the *zeitgeist* at the present moment when we have become acutely aware of how inequitable our treatment system is for racial and ethnic minority individuals and when a world-wide viral pandemic and an explosion of fentanyl in the drug supply have drastically altered the dynamics of the opioid epidemic. This series of papers helps tremendously to point our way forward: we need to learn how to make research and treatment more equitable; we need to invigorate our attempts to get MOUD treatment to youth; we need to assure “no wrong door” so that MOUD becomes available in every possible health care setting; we need to figure out better ways to keep patients in treatment; and we can probably advance all these goals by harnessing technology, including telemedicine, to deliver care in a variety of ways. Despite the successes of the CTN, much work is yet needed to develop effective strategies to: mitigate stigma associated with OUD and its treatment; address co-occurring conditions (e.g., pain, depression, trauma) and other substance use (e.g., stimulant use); and promote linkage to MOUD across different access points (e.g., syringe exchange programs). This collection of papers makes clear that current and forthcoming CTN research will forcefully move us in these directions to confront these and ongoing and evolving challenges.
